# Construction of a 1D/0D/2D BiFeO_3_/Ag/g-C_3_N_4_ Z-scheme heterojunction for enhanced visible light photocatalysis of methylene blue

**DOI:** 10.1039/d5ra04825g

**Published:** 2025-09-02

**Authors:** Donghai Li, Yunrui Xu, Shilin Zhang, Linping Wang

**Affiliations:** a College of Chemical Engineering, Qinghai University Xining-810016 China wanglp@qhu.edu.cn

## Abstract

To improve the utilization of solar energy and the efficiency of photocatalytic organic pollutant degradation, novel Z-scheme heterojunctions with high visible light catalytic performances have been widely developed. Herein, a novel Z-scheme BiFeO_3_/Ag/g-C_3_N_4_ heterojunction with a hierarchical 1D/0D/2D structure and visible light absorption was constructed by matching the suitable band structure between 1D BiFeO_3_ and 2D g-C_3_N_4_ and employing the localized surface plasmon resonance (LSPR) effect of 0D Ag nanoparticles. BiFeO_3_ nanofibers were synthesized *via* the electrospinning technique, providing short electron transport paths and visible light absorption range for g-C_3_N_4_. Plasmonic Ag nanoparticles were photodeposited on the surface of BiFeO_3_ to enhance the separation efficiency of the photogenerated electron–hole pairs between the bulk interfaces. UV-vis DRS, PL, photocurrent and EIS spectra confirmed the important roles of Ag and BiFeO_3_ in improving the photocatalytic activity of the Z-scheme heterojunction. The working principle of the BiFeO_3_/Ag/g-C_3_N_4_ Z-scheme heterojunction was proposed, indicating that ˙O_2_^−^ and ˙OH are the main active species for the photocatalytic degradation of methylene blue (MB). The prepared BiFeO_3_/Ag/g-C_3_N_4_ heterojunction exhibited the highest photocatalytic activity, where its rate constant was 5.84 times higher than that of the pristine BiFeO_3_ and 3.26 times higher than that of the pristine g-C_3_N_4_. This study offers a new means for the design of novel high-performance photocatalysts, which can be a promising candidate in industrial applications.

## Abstract 2

To improve the utilization of solar energy and the efficiency of photocatalytic organic pollutant degradation, novel Z-scheme heterojunctions with high visible light catalytic performances have been widely developed.

## Graphical abstract

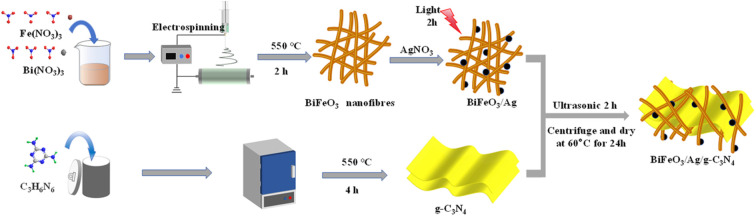

## 1. Introduction

Nowadays, solar-based photocatalytic technology is a promising solution to the global energy crisis and environmental remediation, including photocatalytic hydrogen production, photocatalytic reduction of CO_2_ and photocatalytic degradation of organic pollutants.^1^ Methylene blue (MB), a sulphur-containing cationic dye, is regarded as a common pollutant in wastewater discharged from textile industries.^2–5^ MB poses a carcinogenic risk to humans and many aquatic organisms and affects the balance of the entire aquatic ecosystem.^6^ Photocatalytic degradation of MB is considered a high-efficiency, low-cost and environmentally friendly water treatment technology, which needs high-performance photocatalysts for the decomposition of MB into CO_2_, H_2_O and some harmless organic molecules.^7^ However, single photocatalytic materials, such as TiO_2_, suffer from the drawbacks of low utilization of the visible light, easy recombination of the photogenerated electron–hole pairs, and low photocatalytic activity.^8,9^ As a typical 2D conjugated polymer photocatalyst, g-C_3_N_4_ possesses the advantages of low cost, non-toxicity, and high specific surface area.^10,11^ However, g-C_3_N_4_ can absorb sunlight only at wavelengths below 460 nm and has a low valence band level (*E*_VB_ = 1.50 eV), which is lower than the oxidation potential for the generation of hydroxyl radicals (˙OH), and thus, its efficiency for the photocatalytic degradation of pollutants is significantly weakened.^12^

To enhance the photocatalytic activity of photocatalysts, numerous strategies have been explored, such as defect engineering, ion doping, and heterojunction construction.^13–15^ Among the numerous approaches, heterojunction construction is regarded as a promising strategy due to its merits in promoting photogenerated carrier separation and enhancing the light absorption and stability.^16^ Generally, the interface structures of heterojunctions are composed of two or more different semiconductor materials, which can be divided into type I, type II, and Z-schemes. Compared with type I and type II heterojunctions, Z-scheme heterojunctions enable efficient electron–hole separation with great retention of redox capability. Zheng *et al.* prepared a WO_3_/g-C_3_N_4_ Z-scheme photocatalyst by calcination and hydrothermal treatment to achieve high redox capacity, efficient charge separation and large reaction surface area for the photocatalytic degradation of dodecylmorpholine (DMP), and its degradation efficiency was 73% in 60 min.^17^ However, the electron transfer rate between heterojunction interfaces greatly affects the efficiency for the photocatalytic degradation of pollutants. In this case, the incorporation of metal nanoparticles exhibiting localized surface plasmon resonance (LSPR) within Z-scheme heterojunctions can accelerate the electron transfer rate across the interface and broaden the absorption spectrum to visible light with enhanced the solar energy utilisation. The Schottky junction at the interface between the metal and the semiconductor inhibits the compounding of photogenerated carriers.

As a typical multiferroic perovskite material, BiFeO_3_ exhibits excellent photovoltaic, thermal, specific ferroelectric and magnetic properties. Especially in the field of photocatalysis, its polarization effect leads to band bending, resulting in the effective separation of excitons.^18,19^ In this study, BiFeO_3_ nanofibres with a suitable band gap (*E*_CB_ = 0.51 eV, *E*_VB_ = 2.61 eV, and *E*_g_ = 2.10 eV) are matched with g-C_3_N_4_ (*E*_CB_ = −1.22 eV, *E*_VB_ = 1.50 eV, and *E*_g_ = 2.72 eV) to form an ideal Z scheme heterojunction.^20,21^ The BiFeO_3_ nanofibres with a large aspect ratio can largely shorten the photogenerated electron transfer pathways.^22^ The Ag nanoparticles acted as a direct electron bridge between BiFeO_3_ and g-C_3_N_4_ to construct an efficient Z-scheme heterojunction, which reduced the electron transfer resistance between the interfaces.^23^ The LSPR effect of the Ag nanoparticles further broadened the light absorption range and lowered the Schottky barrier to enhance the solar energy utilization. We achieved the degradation of MB using the 1D/0D/2D BiFeO_3_/Ag/g-C_3_N_4_ Z-scheme heterojunction and proposed its rational mechanism *via* free radical trapping experiments. This study provides new insights into the development of high-performance Z-scheme photocatalysts for the efficient photocatalytic degradation of pollutants in wastewater.

## 2. Experimental

### 2.1. Materials

Bismuth nitrate pentahydrate (Bi(NO_3_)_3_·5H_2_O, 99.9%), melamine (C_3_H_6_N_6_, 99%), iron(iii) nitrate nonahydrate (Fe(NO_3_)_3_·9H_2_O, 99.9%) and polyvinylpyrrolidone (PVP) with an average molecular weight of 1 200 000 were purchased from Shanghai Aladdin Reagent Co., Ltd. Silver nitrate (AgNO_3_, 99.8%) was purchased from Oubokai Chemical Co., Ltd. *N*,*N*-Dimethylformamide (DMF, 99.5%) and acetone (AC, C_3_H_6_O, 99.5%) were purchased from Shanghai Guoyao Group Chemical Reagent Co., Ltd. Anhydrous ethanol (C_2_H_5_OH, 99.7%) and methanol (CH_3_OH, 99.5%) were purchased from Chengdu Chron Chemical Co., Ltd. Methylene blue (C_16_H_18_N_3_ClS, 98.5%) was purchased from Shanghai Zhanyun Chemical Co., Ltd.

### 2.2. Synthesis of g-C_3_N_4_

g-C_3_N_4_ was prepared by pyrolyzing melamine.^24^ In detail, 10 g of melamine powder was initially placed in a lidded ceramic crucible and subjected to heating in a muffle furnace. The furnace was heated at a rate of 5 °C min^−1^ until the temperature reached 550 °C and was maintained at this temperature for 2 h. At the end of the calcination process, the material was cooled naturally to room temperature and ground with a mortar and pestle to obtain the final g-C_3_N_4_ powder.

### 2.3. Synthesis of BiFeO_3_

BiFeO_3_ nanofibres were prepared by the electrospinning method. 1.00 g Bi(NO_3_)_3_·5H_2_O and 0.75 g Fe(NO_3_)_3_·9H_2_O were dissolved in 2.50 mL ultrapure water, and then 1.50 mL glacial acetic acid was added and stirred for 2 h. In parallel, 0.93 g PVP was dissolved in 5.63 mL mixed solution of AC and DMF (AC/DMF = 1 : 2). These two solutions were mixed and stirred for 4 h to obtain a golden-yellow precursor solution for electrostatic spinning. An applied voltage of 16.50 kV and an advancement speed of 0.05 mm min^−1^ were used to obtain the BiFeO_3_ nanofibre precursor. The BiFeO_3_ nanofibre precursor was placed in a muffle furnace and heated to 550 °C for 2 h at a ramp rate of 1 °C min^−1^. The calcined samples were cooled to room temperature in an air environment and placed in an onyx mortar for grinding to obtain pure BiFeO_3_ nanofibres.

### 2.4. Synthesis of BiFeO_3_/Ag_*x*_

The BiFeO_3_/Ag_*x*_ compound was synthesised *via* the photodeposition of Ag nanoparticles on BiFeO_3_ nanofibres by using CH_3_OH as a reducing agent. 0.1 g BiFeO_3_ nanofibres and a certain amount of AgNO_3_ were added to a mixed solution of 10 mL CH_3_OH and 40 mL H_2_O, and sonicated for 10 min. The mixed solution was irradiated by a xenon lamp for 2 h. After irradiation, the mixed solution was centrifuged at 12 000 rpm for 10 min, washed twice with ethanol, and dried under vacuum at 60 °C for 12 h to obtain the BiFeO_3_/Ag_*x*_ samples, where *x* denotes the mass fraction of Ag.

### 2.5. Synthesis of (BiFeO_3_/Ag_*x*_)/(g-C_3_N_4_)_*y*_

**Fig. 1:**
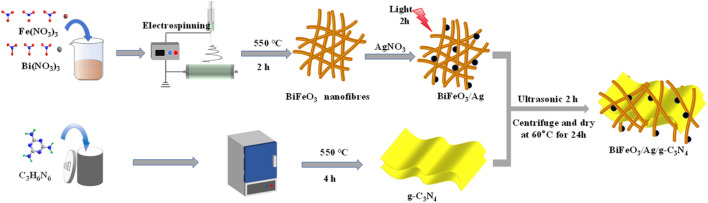
Schematic of the preparation of BiFeO_3_/Ag/g-C_3_N_4_.

0.1 g of synthesised g-C_3_N_4_ was added to 50 mL of ultrapure water and sonicated for 0.5 h to form a homogeneous solution. A certain amount of BiFeO_3_/Ag_*x*_ was dissolved in the solution of g-C_3_N_4_ and ultrasonicated for 2 h. The mixed solution was subsequently centrifuged at 12 000 rpm, and the resulting precipitate was dried under vacuum at 60 °C for 24 h to obtain the (BiFeO_3_/Ag_*x*_)/(g-C_3_N_4_)_*y*_ composites, where the ratio of *y* is defined as the mass fraction of g-C_3_N_4_, as shown in Fig. 1.

### 2.6. Characterization

The morphologies of the samples were characterized using a scanning electron microscope (SEM, JSM-7900F, JEOL). The elemental composition of the samples was characterized using an energy-dispersive X-ray spectrometer (EDX, JSM-7900F, JEOL). The specific surface area of the photocatalyst was determined using a fully automatic specific surface area and porosity analyzer (BET, ASAP 2460, Micromeritics). The phase identification of the samples was performed on a powder X-ray diffractometer (XRD, Ultima IV, Rigaku). Elemental states and surface chemistry were examined by X-ray photoelectron spectroscopy (XPS, Nexsa G2, Thermo Fisher). The absorbance of the pollutants was determined by ultraviolet-visible spectroscopy (UV-vis, UV-2550, Shimadzu). The functional groups of the materials were tested using a Fourier transform infrared spectrometer (FTIR, VERTEX70V, Bruker). The photogenerated electron–hole pair recombination rates were analysed using a photoluminescence spectrometer (PL, Cary Eclipse, Agilent), operating at room temperature with an excitation wavelength of 300 nm. The light absorption performances of the samples were tested using a solid-state ultraviolet-visible diffuse reflectance spectrometer (DRS, Cary 5000, Agilent). The intermediate products of the photocatalytic degradation of MB were determined by liquid chromatography-mass spectrometry (LC-MS, Integrion + TSQ Fortis Plus, Thermo Scientific).

### 2.7. Electrochemical measurements

Photoelectrochemical properties were measured by means of a conventional three-electrode system. Sample-modified indium tin oxide-coated glass was used as the working electrode, a carbon rod as the counter electrode, and a saturated calomel electrode as the reference electrode. In the photocurrent tests, a 0.1 mol L^−1^ ascorbic acid (AA) solution was used as the electrolyte and a 300 W xenon lamp was used as the light source, which was switched on and off at 20 s intervals during the test period of 0–160 s. In the Nyquist plot measurements, a 2.0 mM 1 : 1 K_3_[Fe(CN)_6_]/K_4_[Fe(CN)_6_] and 0.1 mol L^−1^ Na_2_SO_4_ solution was used as the electrolyte with an applied bias voltage of 0.2 V. In the Mott–Schottky tests, a 0.5 mol L^−1^ Na_2_SO_4_ solution was used as the electrolyte.

### 2.8. Photocatalytic activity evaluation

The catalytic performance of the prepared samples was evaluated using MB solution under simulated sunlight (xenon lamp = 300 W). 10 mg catalyst was added to 50 mL of MB solution (*c* = 10 mg L^−1^) at room temperature and magnetically stirred in the dark for 30 min to ensure adsorption and desorption equilibrium. Under light illumination, 2 mL of the suspension was collected and filtered through a 0.45 μm mixed cellulose ester (MCE) membrane. The photocatalytic activity of the catalyst was analyzed by measuring its absorbance at 664 nm on a UV-vis spectrophotometer.

## 3. Results

### 3.1. Morphology and structure analysis

#### 3.1.1. XRD analysis

**Fig. 2:**
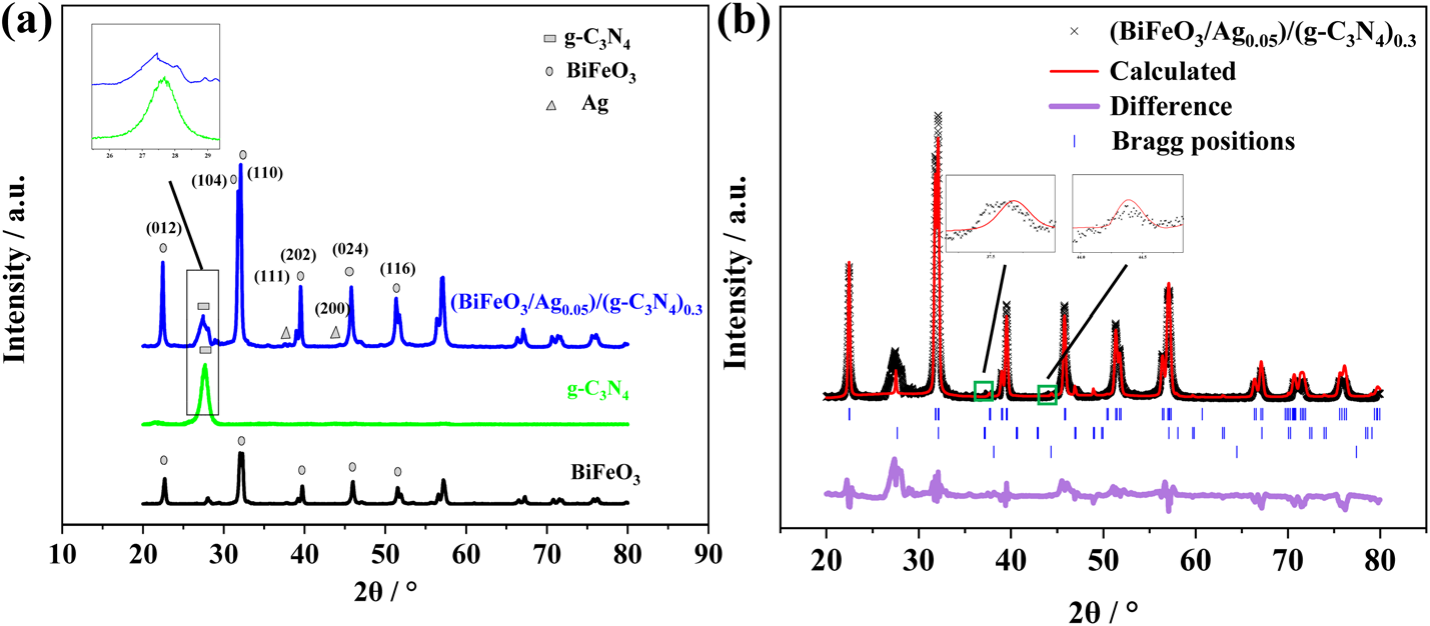
(a) XRD patterns of g-C_3_N_4_, BiFeO_3_, and (BiFeO_3_/Ag_0.05_)/(g-C_3_N_4_)_0.3_. (b) Rietveld refinement XRD patterns of (BiFeO_3_/Ag_0.05_)/(g-C_3_N_4_)_0.3_.

The XRD analysis confirmed the successful preparation of g-C_3_N_4_, BiFeO_3_, and (BiFeO_3_/Ag_0.05_)/(g-C_3_N_4_)_0.3_, as shown in Fig. 2a. The main diffraction peak located at the 2*θ* value of 27.5° is attributed to the (002) crystal plane of g-C_3_N_4_ (JCPDS no. 50-1512).^25^ The main diffraction peaks for the prepared BiFeO_3_ at 2*θ* = 22.5°, 32.1°, 32.3°, 39.8°, 45.8°, and 52.3° correspond to the (012), (104), (110), (202), (024), and (116) crystal planes of BiFeO_3_, respectively (JCPDS no. 86-1518).^26^ Comparatively, (BiFeO_3_/Ag_0.05_)/(g-C_3_N_4_)_0.3_ exhibits the main characteristic peaks of BiFeO_3_, g-C_3_N_4_, and Ag for the (111), (200), and (220) crystal planes at 2*θ* = 37.6°, 44.0° and 63.8°, respectively (JCPDS no. 65-2871).^27^ We performed Rietveld refinement on (BiFeO_3_/Ag_0.05_)/(g-C_3_N_4_)_0.3_. As shown in Fig. 2b, peaks corresponding to the g-C_3_N_4_ phase, BiFeO_3_ phase, and Ag^0^ phase can clearly be observed. By simulating g-C_3_N_4_ (*a* = 6.4505 Å, *b* = 6.4505 Å, *c* = 2.4231 Å and *V* = 87.3171 Å^3^),^28^ BiFeO_3_ (*a* = 5.5881 Å, *b* = 5.5881 Å, *c* = 13.9072 Å and *V* = 376.0994 Å^3^),^29^ and Ag (*a* = 4.0842 Å, *b* = 4.0842 Å, *c* = 4.0842 Å and *V* = 68.1923 Å^3^),^30^ we obtained the lattice parameters and phase composition of the material. As shown in Table 1, the lattice parameters of g-C_3_N_4_ (*a* = 6.4456 Å, *b* = 6.4456 Å, *c* = 2.4206 Å and *V* = 87.1032 Å^3^), BiFeO_3_ (*a* = 5.5786 Å, *b* = 5.5786 Å, *c* = 13.8601 Å and *V* = 373.5562 Å^3^), and Ag (*a* = 4.0683 Å, *b* = 4.0683 Å, *c* = 4.0683 Å and *V* = 68.1923 Å^3^) are almost identical to their standard card values, indicating that the synthesized material has high crystallinity and purity.

**Table 1:** Microstructural parameters obtained from the Rietveld analysis of (BiFeO_3_/Ag_0.05_)/(g-C_3_N_4_)_0.3_

Sample	Phase	Space group	*a* (Å)	*b* (Å)	*c* (Å)	*V* (Å^3^)	*R*_WP_ (%)	*R*_P_ (%)	Volume (%)
	JCPDS no. 50-1512	*P*6_3_/*m*	6.4505	6.4505	2.4231	87.3171	—	—	—
	JCPDS no. 86-1518	*R*3*c*	5.5881	5.5881	13.9072	376.0994	—	—	—
	JCPDS no. 65-2871	*Fm*3̄*m*	4.0842	4.0842	4.0842	68.1923	—	—	—
(BiFeO_3_/Ag_0.05_)/(g-C_3_N_4_)_0.3_	g-C_3_N_4_	*P*6_3_*cm*	6.4456	6.4456	2.4206	87.1032	1.20	0.82	33.1
	BiFeO_3_	*R*3*c*	5.5786	5.5786	13.8601	373.5562			65.1
	Ag	*Fm*3̄*m*	4.0683	4.0683	4.0683	68.1923			1.8

#### 3.1.2. SEM analysis

**Fig. 3:**
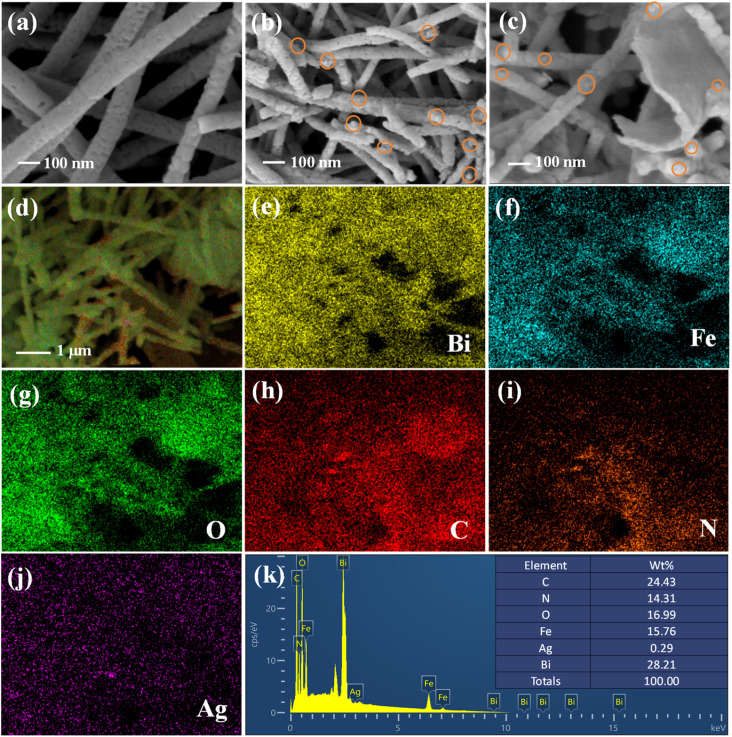
SEM images of (a) BiFeO_3_, (b) BiFeO_3_/Ag_0.05_, and (c) (BiFeO_3_/Ag_0.05_)/(g-C_3_N_4_)_0.3_ and (d–k) EDX element mapping of (BiFeO_3_/Ag_0.05_)/(g-C_3_N_4_)_0.3_.

The micromorphology of the catalysts was investigated by SEM. As shown in Fig. 3a, BiFeO_3_ exhibits a fibrous structure that is continuous and uniformly dispersed with a diameter of around 100 nm. The Ag nanoparticles are decorated on the BiFeO_3_ nanofibers with a particle size in the range of 10 to 20 nm (Fig. 3b). Non-uniform broken BiFeO_3_ nano-fibers are formed due to the photodeposition of Ag nanoparticles on BiFeO_3_. After the combination of BiFeO_3_/Ag with g-C_3_N_4_, flake-like g-C_3_N_4_ appeared in the nanofibers (Fig. 3c). The 1D/0D/2D BiFeO_3_/Ag/g-C_3_N_4_ multidimensional structure increases the specific surface area of the single materials, which was further confirmed by the N_2_ adsorption–desorption isotherm tests. The N_2_ adsorption–desorption isotherm curves are displayed in Fig. S1, and the specific surface area and pore volume values are displayed in Table S1. The surface area of (BiFeO_3_/Ag_0.05_)/(g-C_3_N_4_)_0.3_ is obviously higher than that of g-C_3_N_4_ and BiFeO_3_/Ag, facilitating the greater exposure of reactive sites to further improve the efficient ion transport and photocatalytic degradation ability. The EDX analysis confirms the presence of Bi, Fe, O, C, N and Ag elements in the (BiFeO_3_/Ag_0.05_)/(g-C_3_N_4_)_0.3_ composite, indicating the successful preparation of the BiFeO_3_/Ag/g-C_3_N_4_ heterojunction (Fig. 3d–k). The presence of Bi, Fe, O, C, N and Ag elements evenly distributed with mass ratios of 28.21%, 15.76%, 16.99%, 24.43%, 14.31%, and 0.29%, respectively, indicates the successful incorporation of a certain amount of Ag in the composite.

#### 3.1.3. FTIR analysis

**Fig. 4:**
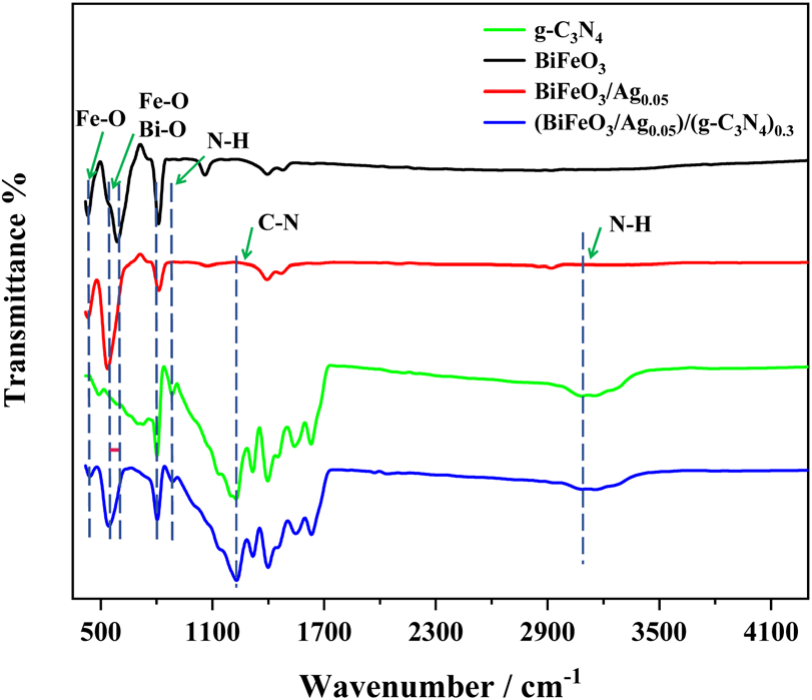
FT-IR spectra of g-C_3_N_4_, BiFeO_3_, BiFeO_3_/Ag_0.05_, and (BiFeO_3_/Ag_0.05_)/(g-C_3_N_4_)_0.3_.

FTIR spectroscopy was employed to determine the surface functional groups in g-C_3_N_4_, BiFeO_3_, BiFeO_3_/Ag_0.05_, and (BiFeO_3_/Ag_0.05_)/(g-C_3_N_4_)_0.3_, as shown in Fig. 4. In the spectrum of g-C_3_N_4_, the absorption peaks at 805 cm^−1^, 887 cm^−1^, 1230 cm^−1^, and 3140 cm^−1^ are attributed to the triazine breathing mode, the deformation mode of N–H, and the stretching vibration of the C–N and N–H bond, respectively.^31–34^ The peaks at 440 cm^−1^ and 544 cm^−1^ are attributed to the bending vibrations of Fe–O in the octahedral FeO_6_ groups, and the stretching vibrations of Fe–O and Bi–O.^35^ After Ag nanoparticles were deposited on the surface of BiFeO_3_, a blue-shift in the absorption peak was observed from 544 cm^−1^ to 538 cm^−1^.^36^ The spectrum of (BiFeO_3_/Ag_0.05_)/(g-C_3_N_4_)_0.3_ contains peaks related to g-C_3_N_4_, and two characteristic BiFeO_3_/Ag_0.05_ peaks at 440 and 538 cm^−1^, confirming the successful fabrication of the heterojunction structure.

#### 3.1.4. XPS analysis

**Fig. 5:**
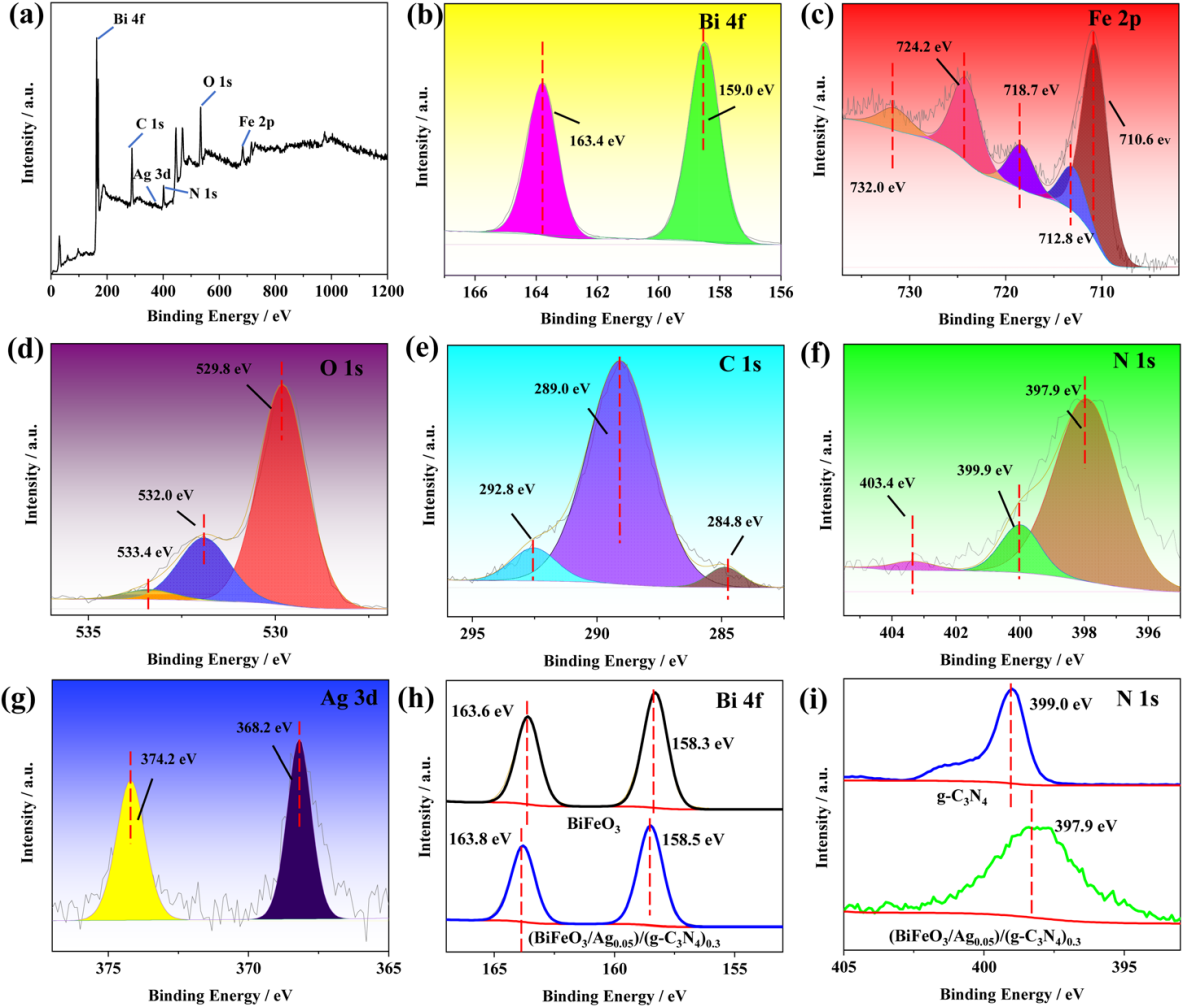
(a) XPS spectrum of (BiFeO_3_/Ag_0.05_)/(g-C_3_N_4_)_0.3_. (b–g) Narrow-scan XPS of (BiFeO_3_/Ag_0.05_)/(g-C_3_N_4_)_0.3_ in the regions of Bi 4f, Fe 2p, O 1s, C 1s, N 1s, and Ag 3d core levels, respectively. (h) Comparison of the XPS spectra of BiFeO_3_ and (BiFeO_3_/Ag_0.05_)/(g-C_3_N_4_)_0.3_ for Bi 4f. (i) Comparison of the XPS spectra of g-C_3_N_4_ and (BiFeO_3_/Ag_0.05_)/(g-C_3_N_4_)_0.3_ for N 1s.

To further pursue the chemical state and composition of the elements in the as-prepared (BiFeO_3_/Ag_0.05_)/(g-C_3_N_4_)_0.3_, its XPS spectrum was recorded, as shown in Fig. 5. Fig. 5a shows the main elements of Fe, Bi, O, N, C and Ag in (BiFeO_3_/Ag_0.05_)/(g-C_3_N_4_)_0.3_. Fig. 5b–g represent the high-resolution XPS spectra of Bi 4f, Fe 2p, O 1s, C 1s, N 1s and Ag 3d, respectively. The Bi 4f spectrum exhibits doublet peaks at 163.4 eV and 159.0 eV, corresponding to Bi 4f_5/2_ and Bi 4f_7/2_ of Bi^3+^, respectively.^37^ The Fe 2p spectrum of (BiFeO_3_/Ag_0.05_)/(g-C_3_N_4_)_0.3_ exhibits three peaks at 710.7 eV, 712.8 eV, and 724.2 eV, which correspond to Fe^2+^ 2p_3/2_, Fe^3+^ 2p_3/2_, and Fe^3+^ 2p_1/2_, respectively. The other peaks centered at 732.0 eV and 718.7 eV are designated as Fe^3+^ satellite peaks.^38,39^ The XPS O 1s spectrum displays three peaks at 529.8 eV, 532.0 eV and 533.4 eV, corresponding to the surface lattice oxygen, surface hydroxyl groups and surface adsorbed oxygen.^40^ In the C 1s XPS spectrum, the peak at 289.0 eV is typically associated with the N–C=N in the triazine units, the peak at 284.8 eV is attributed to C–O species on the surface of g-C_3_N_4_, and the peak at 292.8 eV can be assigned to π electronic excitation.^41,42^ In the XPS N 1s spectrum, the peak at 397.9 eV is typically attributed to C–N=C, and the peak at 399.9 eV corresponds to N–H.^42^ Another peak centered at 403.4 eV is related to pi-excitations on heterocycles.^43^ In the Ag 3d XPS spectrum, the peaks at 374.2 eV and 368.2 eV are attributed to Ag 3d_5/2_ and Ag 3d_3/2_, respectively. The difference between these two peaks is 6 eV, indicating that silver atoms are in a metallic state.^44^ To further confirm the electron transfer in the heterojunction, the peak positions of Bi 4f in BiFeO_3_, and N 1s of g-C_3_N_4_ were examined by XPS. As shown in Fig. 5h and i, compared with BiFeO_3_ and g-C_3_N_4_, the position of Bi 4f in (BiFeO_3_/Ag_0.05_)/(g-C_3_N_4_)_0.3_ shifted positively by approximately 0.2 eV, while the position of N 1s shifted negatively by approximately 1.1 eV. Similarly, as shown in Fig. 5g, the Ag 3d peak in (BiFeO_3_/Ag_0.05_)/(g-C_3_N_4_)_0.3_ exhibits a positive shift of approximately 0.2 eV relative to the reported values for Ag^0^ (368.0 and 374.0 eV).^45^ The peak shifting in (BiFeO_3_/Ag_0.05_)/(g-C_3_N_4_)_0.3_ demonstrates that electrons could migrate from BiFeO_3_ to Ag, and then to g-C_3_N_4_, resulting a fixed Z-scheme heterojunction photocatalytic system at the double interfaces of BiFeO_3_/Ag and g-C_3_N_4_/Ag.^46^

#### 3.1.5. UV-vis DRS and PL analysis

**Fig. 6:**
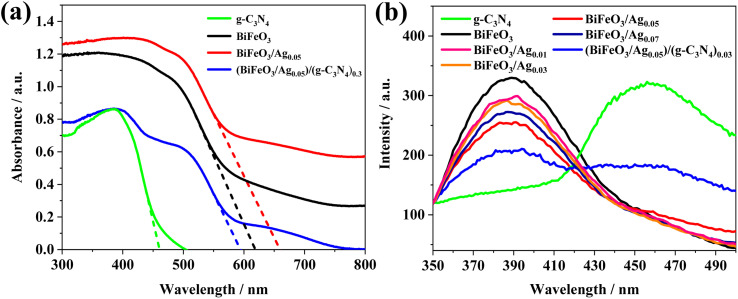
(a) UV-vis DRS spectra of the as-prepared g-C_3_N_4_, BiFeO_3_, BiFeO_3_/Ag_0.05_ and (BiFeO_3_/Ag_0.05_)/(g-C_3_N_4_)_0.3_. (b) PL spectra of g-C_3_N_4_, BiFeO_3_, BiFeO_3_/Ag_(0.01–0.07)_, and (BiFeO_3_/Ag_0.05_)/(g-C_3_N_4_)_0.3_ (300 nm excitation).

The UV-vis absorbance of g-C_3_N_4_, BiFeO_3_, BiFeO_3_/Ag_0.05_ and (BiFeO_3_/Ag_0.05_)/(g-C_3_N_4_)_0.3_ was tested using UV-visible diffuse reflectance spectroscopy. As shown in Fig. 6a, g-C_3_N_4_ has narrow visible light absorption ability, and the absorption edge of g-C_3_N_4_ is located at 458 nm. The pure-phase BiFeO_3_ exhibits strong visible light absorption properties in the range of 400–550 nm, and its absorption boundary is located at 619 nm. After loading Ag nanoparticles, the absorption edge of BiFeO_3_/Ag_0.05_ is markedly red-shifted to 660 nm. Due to the LSPR effect of the Ag nanoparticles, ‘hot’ electrons were injected into the BiFeO_3_ interface, further broadening the light absorption range and lowering the Schottky barrier.^47^ The LSPR effect is largely dependent on the size and shape of the Ag nanoparticles, and the LSPR peaks could be tuned to ∼450 nm based on the size of Ag nanoparticles in the range of 10–20 nm.^48,49^ The (BiFeO_3_/Ag_0.05_)/(g-C_3_N_4_)_0.3_ heterojunction has an excellent visible light absorption performance with an absorption edge at 594 nm.

PL spectra were used to evaluate the separation rate of photogenerated electron–hole pairs. As shown in Fig. 6b, the PL emission intensity of BiFeO_3_/Ag is markedly reduced than that of pristine BiFeO_3_. This indicates that the Ag nanoparticles deposited on the surface of BiFeO_3_ formed a Schottky junction, which can suppress the recombination of electron–hole pairs at the interface of BiFeO_3_/Ag. In addition, BiFeO_3_/Ag_0.05_ exhibits the lowest intensity, and the intensity of its PL emission peak decreases with an increase in the concentration of Ag nanoparticles. Given that excessive Ag nanoparticles may accumulate, resulting in the formation of composite centers, the separation rate of photogenerated electron–hole pairs decreases.^47^ The PL intensity of (BiFeO_3_/Ag_0.05_)/(g-C_3_N_4_)_0.3_ was significantly reduced compared with that of pure BiFeO_3_ and g-C_3_N_4_, indicating that the separation efficiency of the photogenerated electron–hole pairs was higher than that of pure BiFeO_3_ and g-C_3_N_4_.

#### 3.1.6. Photocurrent and EIS analysis

**Fig. 7:**
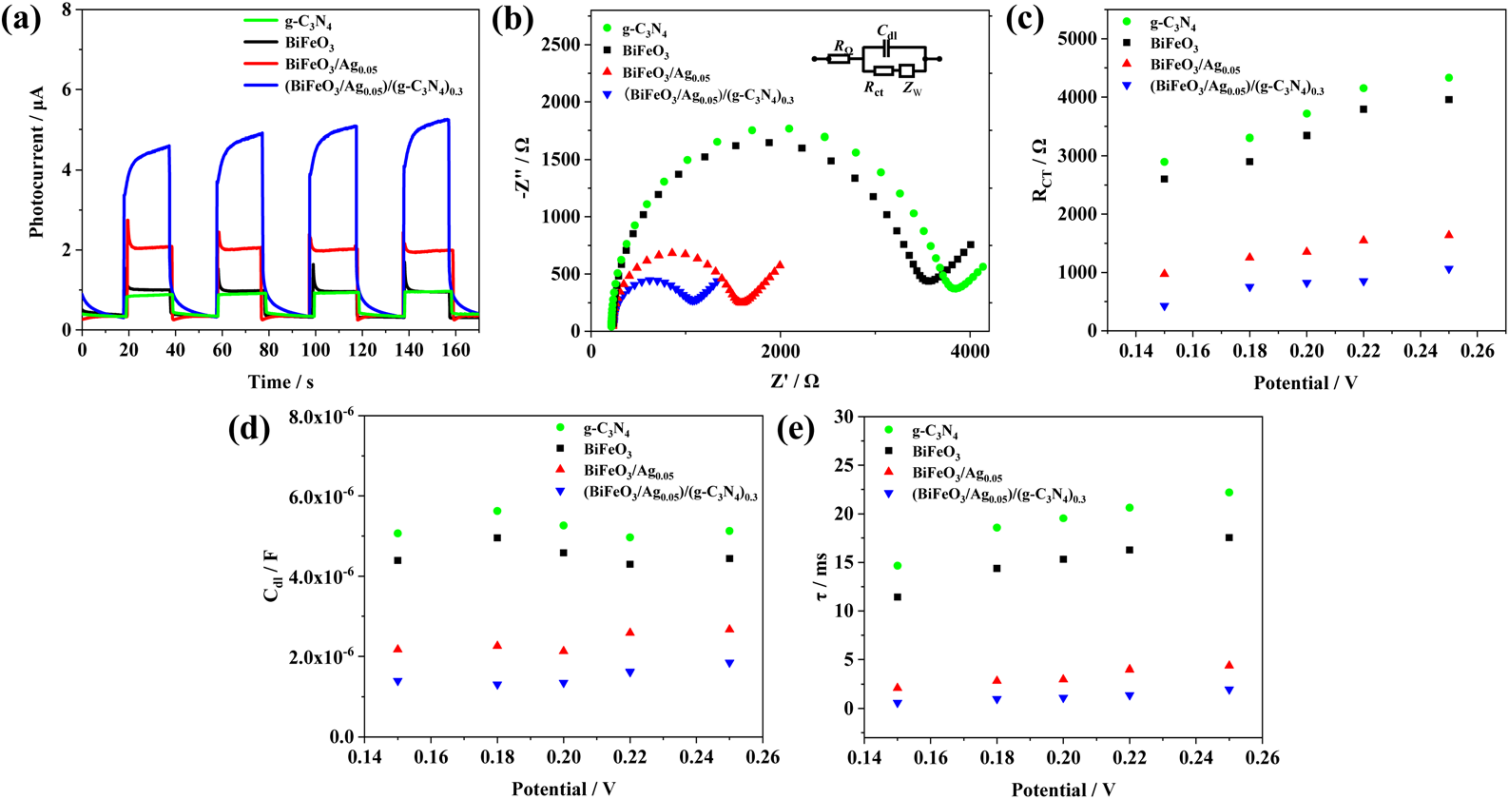
(a) Photocurrent spectra of g-C_3_N_4_, BiFeO_3_, BiFeO_3_/Ag_0.05_, and (BiFeO_3_/Ag_0.05_)/(g-C_3_N_4_)_0.3_. (b) EIS spectra of g-C_3_N_4_, BiFeO_3_, BiFeO_3_/Ag_0.05_ and (BiFeO_3_/Ag_0.05_)/(g-C_3_N_4_)_0.3_, inset: equivalent circuit (photocurrent responses measured at a bias voltage of 0.0 V (*vs.* SCE) in 0.1 M ascorbic acid and EIS responses measured in 0.1 M Na_2_SO_4_ containing 2.0 mM 1 : 1 K_3_[Fe(CN)_6_]/K_4_[Fe(CN)_6_] after biasing at 0.2 V (*vs.* SCE) for 200 s). Impedance fitting results of (c) *R*_ct_ and (d) *C*_dl_ data *versus* the applied potential. (e) Electron lifetimes of the photocatalysts *versus* applied potential.

Transient photocurrent measurements were performed to investigate the photogenerated carrier separation and interfacial charge transfer efficiency of the samples. As shown in Fig. 7a, the photocurrent intensity of g-C_3_N_4_ is 0.61 μA, while that of BiFeO_3_ is 0.65 μA. With the addition of Ag nanoparticles, the photocurrent intensity of BiFeO_3_/Ag_0.05_ significantly increased to 1.65 μA, which is attributed to the good conductive ability of the Ag nanoparticles. The photocurrent intensity of (BiFeO_3_/Ag_0.05_)/(g-C_3_N_4_)_0.5_ is 4.62 μA, which is about 7.57-fold higher than that of g-C_3_N_4_ and 7.10-fold higher than that of BiFeO_3_. The heterojunction structure of (BiFeO_3_/Ag_0.05_)/(g-C_3_N_4_)_0.3_ provides a more efficient transport path for photogenerated electrons by the synergistic effect of LSPR Ag, BiFeO_3_ and g-C_3_N_4_.

Electrochemical impedance spectroscopy (EIS) of the g-C_3_N_4_, BiFeO_3_, BiFeO_3_/Ag_0.05_, and (BiFeO_3_/Ag_0.05_)/(g-C_3_N_4_)_0.3_ modified ITO electrodes was also carried out to evaluate the kinetics of interfacial charge transmission and fitted to the circuit equivalent, the Randles and Ershler model (inset of Fig. 7b).^50^ As shown in Fig. 7b, g-C_3_N_4_ exhibits the largest semicircle with the charge transfer resistance (*R*_ct_) value of about 3694 Ω, indicating that g-C_3_N_4_ possesses poor electron conductive ability. Compared to BiFeO_3_ (3113 Ω), BiFeO_3_/Ag_0.05_ has a significantly lower electrochemical impedance with an *R*_ct_ value of 1350 Ω for the rapid transfer of carriers by LSPR Ag and formed Schottky junctions. The small amount of Ag loading can significantly lower the charge transfer resistance and exhibit better photogenerated carrier mobility. The (BiFeO_3_/Ag_0.05_)/(g-C_3_N_4_)_0.3_ heterojunction exhibits a significantly reduced arc radius than that of the other materials with an *R*_ct_ value of 825 Ω. The lower charge transfer resistance of the (BiFeO_3_/Ag_0.05_)/(g-C_3_N_4_)_0.3_ heterojunction implies a faster electron transfer rate and smaller electron–hole recombination effect. In addition, the EIS equivalent circuit can be used to evaluate the charge-transfer abilities of photoelectrochemical systems, where the double-layer capacitance (*C*_dl_) is the double layer charge storage capacity at the semiconductor/electrolyte interface and *R*_ct_ is the charge-transfer resistance at the same interface.^51^ To verify the superior photo-generated carrier separation performance of (BiFeO_3_/Ag_0.05_)/(g-C_3_N_4_)_0.3_, EIS measurements were carried out by varying the potential from 0.15 V to 0.25 V (*versus* saturated calomel electrode (SCE)) in the dark. Fitting to the circuit equivalent, the *R*_ct_ and *C*_dl_ parameters of the different photocatalysts were obtained, as displayed in Fig. 7c and d, respectively. The electron lifetime (*τ*) at the depletion layer of the photocatalysts was calculated according to eqn (1), as follows:^52^

1 *τ* = *R*_ct_*C*_dl_

Fig. 7e presents the electron lifetime of g-C_3_N_4_, BiFeO_3_, BiFeO_3_/Ag_0.05_, and (BiFeO_3_/Ag_0.05_)/(g-C_3_N_4_)_0.3_, where a shorter lifetime means faster charge transfer ability given that the amount of electrons in the depletion layer of the photocatalysts is very low. The electron lifetime follows the order of g-C_3_N_4_ > BiFeO_3_ > BiFeO_3_/Ag_0.05_ > (BiFeO_3_/Ag_0.05_)/(g-C_3_N_4_)_0.3_. The Z-scheme (BiFeO_3_/Ag_0.05_)/(g-C_3_N_4_)_0.3_ photocatalytic system has superior photogenerated electron–hole pair separation ability, retaining a greater number of photo-generated carriers for producing radicals, which will enhance the photocatalytic efficiency for the degradation of pollutants.

### 3.2. Evaluation of photocatalytic activity

#### 3.2.1. Efficiency of photocatalytic degradation of MB

The photocatalytic activity of the (BiFeO_3_/Ag_0.05_)/(g-C_3_N_4_)_0.3_ heterojunction was assessed by the photocatalytic degradation of MB and calculated using eqn (2), as follows:^53^

**Fig. 8:**
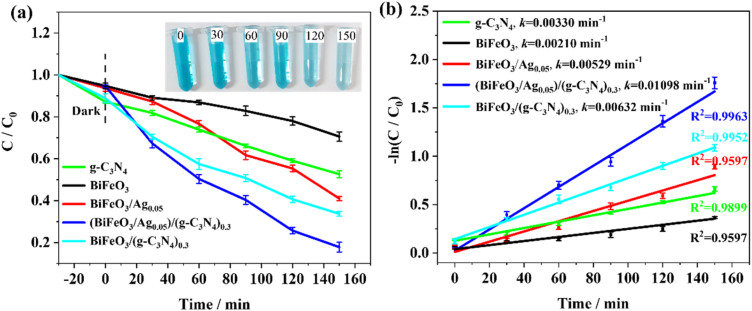
(a) Catalytic efficiency (inset: the variation of MB solution color by time); (b) catalytic kinetic plots of g-C_3_N_4_, BiFeO_3_, BiFeO_3_/Ag_0.05_, (BiFeO_3_/Ag_0.05_)/(g-C_3_N_4_)_0.3_ and BiFeO_3_/(g-C_3_N_4_)_0.3_ for MB (catalyst = 10 mg, MB = 10 mg L^−1^, xenon lamp = 300 W).

2 Degradation% = 1 − *A*/*A*_0_ = 1 − *C*/*C*_0_

where *A*_0_ is the initial absorbance of the MB solution, *A* is the final absorbance of the MB solution after illumination, *C*_0_ is the initial concentration of MB solution, and *C* is the final concentration of MB solution after illumination. Before the photocatalytic degradation of MB, adsorption equilibrium occurs for 30 min in the dark reaction, and the maximum adsorption of MB reached 13% degradation by g-C_3_N_4_, while (BiFeO_3_/Ag_0.05_)/(g-C_3_N_4_)_0.3_ caused 6% degradation, indicating that g-C_3_N_4_ had stronger dark adsorption ability (Fig. 8a). The corresponding photocatalytic kinetic studies proved that the dark reactions followed a pseudo-second-order kinetic reaction (Fig. S2 and S3). The adsorption kinetic studies were evaluated using eqn (3) and (4), as follows:^54^

3 ln(*q*_e_ − *q*) = ln *q*_e_ − *k*_1_*t*

4 *t*/*q* = 1/(*k*_2_*q*_e_^2^) + *t*/*q*_e_

where *q*_e_ and *q* are the adsorption capacities at equilibrium and at time *t* and *k*_1_ and *k*_2_ are rate constants for the pseudo-first-order and pseudo-second-order kinetics, respectively. In the case of g-C_3_N_4_, the pseudo-second-order kinetic model (*R*^2^ = 0.9948) provides a better fit than the pseudo-first-order kinetic model (*R*^2^ = 0.9002). Similarly, (BiFeO_3_/Ag_0.05_)/(g-C_3_N_4_)_0.3_ was also significantly better fitted by the pseudo-second-order kinetic model (*R*^2^ = 0.9802) than the pseudo-first-order kinetic model (*R*^2^ = 0.7908). Therefore, both g-C_3_N_4_ and (BiFeO_3_/Ag_0.05_)/(g-C_3_N_4_)_0.3_ follow the pseudo-second-order kinetic model during the dark reaction periods. After 40 min of dark adsorption and 150 min illumination, the maximum removal efficiency of MB reached 84.60% by the (BiFeO_3_/Ag_0.05_)/(g-C_3_N_4_)_0.3_ heterojunction, while that of g-C_3_N_4_, BiFeO_3_, BiFeO_3_/Ag_0.05_, and BiFeO_3_/(g-C_3_N_4_)_0.3_ was only 49.2%, 31.3%, 60.0% and 66.3%, respectively. The corresponding UV-vis spectra are shown in Fig. S4, and the MB degradation efficiency was calculated at the maximum absorption wavelength of 664 nm. In addition, the optimum mass ratio of BiFeO_3_/Ag/g-C_3_N_4_ in photocatalytic MB degradation was investigated, as shown in Fig. S5. According to Fig. S5a, the BiFeO_3_/Ag composite achieved the best catalytic effect when the amount of Ag is 5 wt%. Additionally, with a mass fraction of g-C_3_N_4_ at 30 wt%, the BiFeO_3_/Ag/g-C_3_N_4_ composite showed the best catalytic effect (Fig. S5b). The photodegradation rate constant was fitted by the first-order reaction kinetics using eqn (5), as follows:^55^

5 ln(*C*/*C*_0_) = −*kt*

where *k* is the photodegradation rate constant. As shown in Fig. 8b, the rate constant of (BiFeO_3_/Ag_0.05_)/(g-C_3_N_4_)_0.3_ (*k* = 0.01163 min^−1^) is 5.84-times than that of BiFeO_3_ and 3.26-times than that of g-C_3_N_4_, demonstrating its superior photocatalytic activity towards MB. The MB removal efficiency of BiFeO_3_/Ag/g-C_3_N_4_ is 2.7-times than that of pure BiFeO_3_ and 1.7-times than that of pure g-C_3_N_4_, confirming the advantages of the 1D/0D/2D layered architecture and synergistic effect in the BiFeO_3_/Ag/g-C_3_N_4_ Z-scheme heterojunction. (i) The 1D/0D/2D multilayered architecture of BiFeO_3_/Ag/g-C_3_N_4_ significantly enhanced the specific surface area of single materials, which could provide more accessible surface sites for the adsorption and degradation of MB. (ii) The LSPR enhancement effect of Ag offers the advantages of low cost, improved visible-light absorption and photoelectron transfer.^56^ In addition, Ag acts as an electronic bridge, promoting electron transfer between BiFeO_3_ and g-C_3_N_4_. (iii) The formation of a Z-scheme heterojunction can retain strong redox potentials by generating holes in the VB of BiFeO_3_ and electrons in the CB of g-C_3_N_4_, and simultaneously form ˙O_2_^−^ and ˙OH radicals to directly react with pollutants. Table S2 presents a comparison with other semiconductor photocatalysts for the degradation of pollutants. The present BiFeO_3_/Ag/g-C_3_N_4_ Z scheme heterojunction displayed an appropriate photo-degradation efficiency, further providing a green and simple method for the construction of visible-light Z-scheme heterojunctions.

#### 3.2.2. Reusability analysis

The photochemical stability and reusability of photocatalysts are key indicators for assessing their catalytic efficiency and long-term performance in practical applications. In this study, the reusability of the (BiFeO_3_/Ag_0.05_)/(g-C_3_N_4_)_0.3_ heterojunction was evaluated, as shown in Fig. S6. The degradation efficiency of (BiFeO_3_/Ag_0.05_)/(g-C_3_N_4_)_0.3_ remained 70.3% after four photocatalytic degradation cycling experiments, indicating its satisfactory photocatalytic stability and reusability for the degradation of MB in water.

#### 3.2.3. Photocatalytic mechanism

**Fig. 9:**
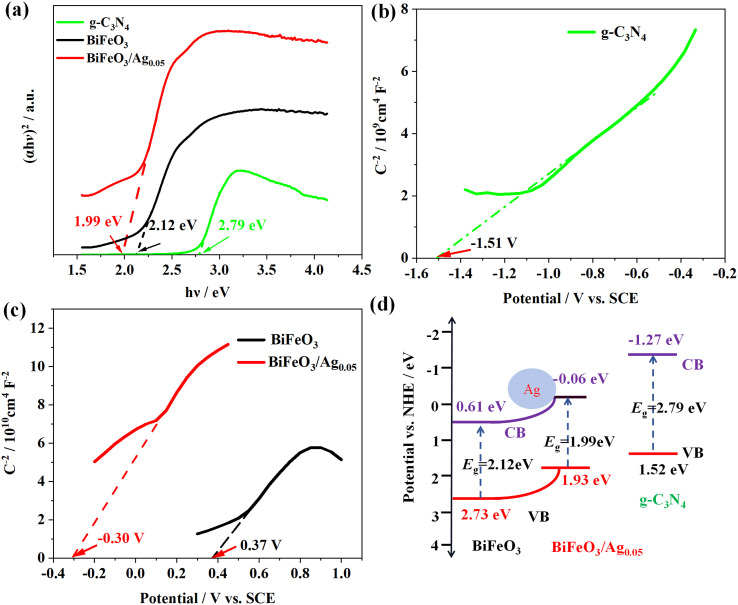
(a) Plots of (*αhν*)^2^*versus hν* for g-C_3_N_4_, BiFeO_3_ and BiFeO_3_/Ag. (b and c) Mott–Schottky plots of pure g-C_3_N_4_ and BiFeO_3_ and BiFeO_3_/Ag, respectively. (The Mott–Schottky effect was measured in a 0.5 M Na_2_SO_4_ solution at 1.0 kHz.) (d) Schematic of the band structures of g-C_3_N_4_, BiFeO_3_ and BiFeO_3_/Ag.

The bandgap of different photocatalysts can be calculated using the UV-vis DRS and Mott–Schottky methods. Fig. 9a presents the plots of (*αhν*)^2^*versus hν* for g-C_3_N_4_, BiFeO_3_ and BiFeO_3_/Ag. The bandgap energies of g-C_3_N_4_, BiFeO_3_ and BiFeO_3_/Ag are determined to be 2.79 eV, 2.12 eV, and 1.99 eV, respectively, which are consistent with the literature values.^57^ As shown in Fig. 9b and c, the flat-band potentials (*V*_fb_) of g-C_3_N_4_, BiFeO_3_ and BiFeO_3_/Ag are −1.51 V, 0.37 V and −0.30 V *versus* SCE, respectively. These flat band potential values are modulated to the normal hydrogen electrode (NHE) values using eqn (6), as follows:^58^

6 *V*_fb_(NHE, pH = 7) = *V*_fb_(SCE) + 0.2415 − 0.059 (7 – pH of the electrolyte)

It has been reported that the conduction band position (*E*_CB_) of n-type semiconductors is very close to *V*_fb_; therefore, the *E*_CB_ values of g-C_3_N_4_, BiFeO_3_ and BiFeO_3_/Ag are −1.27 eV, 0.61 eV and −0.06 eV, respectively.^59^ The valence-band edge (*E*_VB_) was subsequently calculated using eqn (7), as follows:

7 *E*_g_ = *E*_VB_ − *E*_CB_

The band structure diagram of the materials is shown in Fig. 9d. The CB and VB potentials of BiFeO_3_/Ag are more negative than that of BiFeO_3_, and thus the accumulated photoelectrons on the CB of BiFeO_3_ easily flow to the Ag nanoparticles. Owing to the lower Fermi level of the Ag nanoparticles, Ag acted as an electron reservoir, surmounting the Schottky barriers at the BiFeO_3_/Ag interface. The LSPR effect of the Ag nanoparticles induced by the oscillation of their surface electrons could further enhance the visible light harvesting of photocatalysts and promote electron–hole separation.^60,61^

**Fig. 10:**
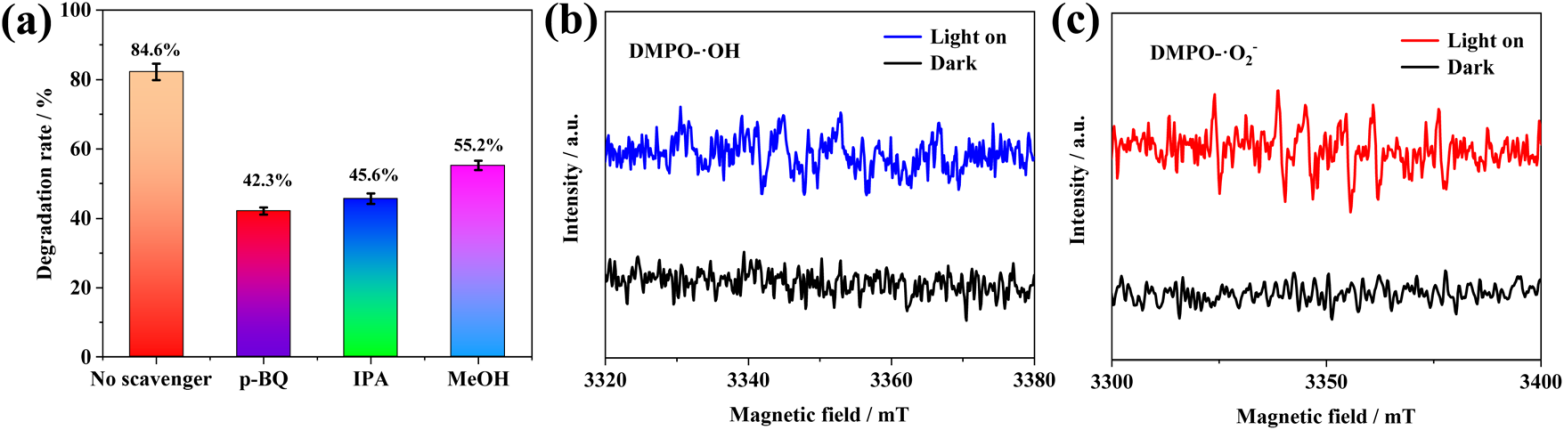
(a) Effect of scavengers on the photocatalytic removal of MB by (BiFeO_3_/Ag_0.05_)/(g-C_3_N_4_)_0.3_ (scavengers' content: 30 mM *p*-BQ, 30 mM IPA, and 30 mM MeOH). EPR spectra of (BiFeO_3_/Ag_0.05_)/(g-C_3_N_4_)_0.3_ for (b) DMPO–˙OH and (c) DMPO–˙O_2_^−^.

As shown in Fig. 10a, in the photocatalytic systems, *p*-benzoquinone (*p*-BQ), isopropanol (IPA), and methanol (MeOH) were added to the MB solution to capture the superoxide (˙O_2_^−^), ˙OH, and h^+^, respectively.^62–64^ After adding IPA and *p*-BQ, separately, the degradation efficiency of MB was severely suppressed, indicating that ˙OH and ˙O_2_^−^ play a major role in the degradation process compared to h^+^, respectively. To ensure effective quenching of target free radicals, we investigated the effect of different scavenger concentrations on the photocatalytic degradation of MB (Fig. S7). As the scavenger concentration increased from 10 mM to 30 mM, the free radical scavenging efficiency remained nearly constant, and thus we selected a scavenger concentration of 30 mM for comparison.

Electron paramagnetic resonance (EPR) experiments were conducted for (BiFeO_3_/Ag_0.05_)/(g-C_3_N_4_)_0.3_ to further verify the production of ˙OH and ˙O_2_^−^. As shown in Fig. 10b and c, no EPR signals were detected in the dark. The EPR signals of ˙OH and ˙O_2_^−^ can be clearly seen under light irradiation, which is consistent with the results of the active species scavenging experiments. This suggests that (BiFeO_3_/Ag_0.05_)/(g-C_3_N_4_)_0.3_ can produce ˙OH and ˙O_2_^−^ radicals, confirming the hypothesis of constructing a Z-scheme photocatalytic system.

**Fig. 11:**
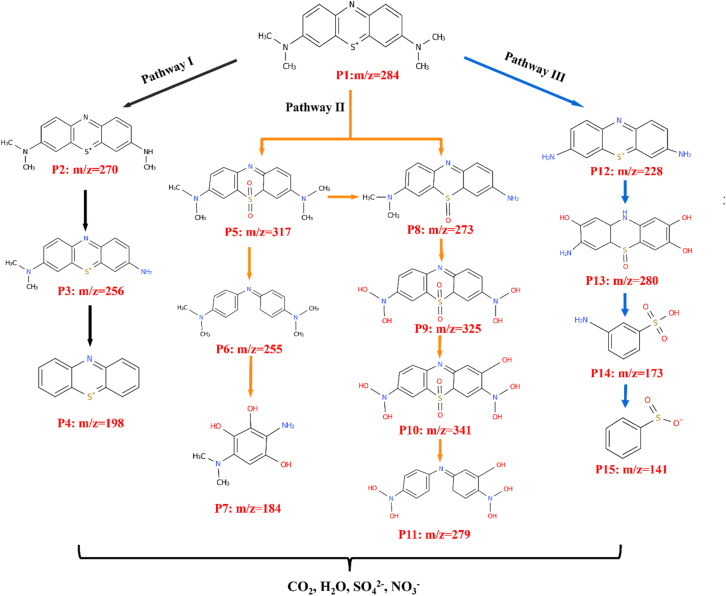
Possible photocatalytic degradation pathways of MB.

Furthermore, the main intermediates yielded during the photocatalytic degradation of MB and the possible degradation pathways were investigated using liquid chromatography-mass spectrometry (LC-MS). According to the *m*/*z* ratios obtained in positive mode, a total of 15 dominant structures was identified under 0, 30, 90, 150 min light irradiation, as shown in Fig. S8, and the possible three degradation pathways under ˙OH and ˙O_2_^−^ induction are shown in Fig. 11. The mass spectrum of MB shows a prominent molecular ion peak at *m*/*z* = 284, which is consistent with a previous report.^65^ The ˙OH and ˙O_2_^−^ radicals may further cause the MB degradation pathways to proceed through demethylation, oxidative, and ring opening to the final fragmentation, and eventually mineralization was achieved.^66,67^ In pathway I, the original MB is demethylated to form intermediate P2 (*m*/*z* = 270), followed by the loss of another methyl group to form P3 (*m*/*z* = 256), and finally converted to the product P4 (*m*/*z* = 198) through demethylation and deamination. In pathway II, the MB molecule is converted to two oxidative products, P5 (*m*/*z* = 317) and P8 (*m*/*z* = 273), by the formation of a sulfoxide group and demethylation process. According to the easily broken bond of N–CH_3_, and the C–N or C–S bond on the central heterocycle of MB, the P5 and P8 intermediates can be degraded into small forms by desulfurization, demethylation, hydroxylation reactions.^68^ In pathway III, the MB molecule is demethylated to form P12 (*m*/*z* = 228), subsequently converting to P13 (*m*/*z* = 280) by the oxidation of the C–S bond on the central heterocycle to sulfoxide, which is further converted to P14 (*m*/*z* = 173) and P15 (*m*/*z* = 141) through the cleavage of the central ring. Eventually, the short open ring structures were completely mineralized into CO_2_, H_2_O, SO_4_^2−^, NO_3_^−^ and some inorganic compounds.^69^

**Fig. 12:**
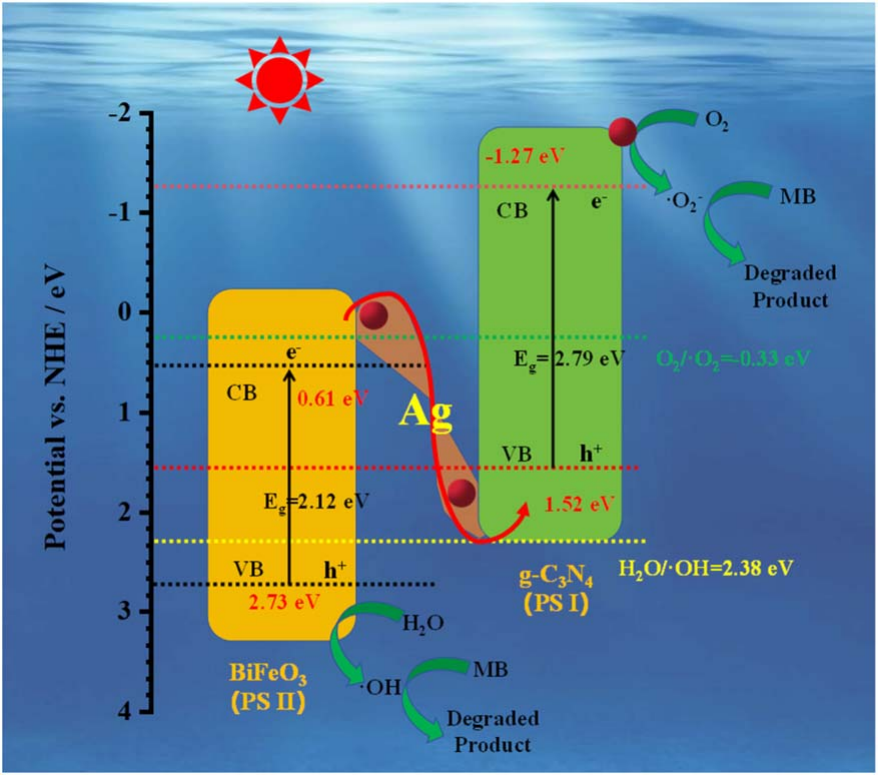
Possible photocatalytic mechanism of the Z-scheme (BiFeO_3_/Ag_0.05_)/(g-C_3_N_4_)_0.3_ system.

Considering the radical scavenging experiments and the bandgap configuration of the prepared g-C_3_N_4_ and BiFeO_3_, a possible photocatalytic mechanism is proposed, as shown in Fig. 12. g-C_3_N_4_ is unable to produce ˙OH (its *E*_VB_ is more negative than 2.38 eV), while BiFeO_3_ is unable to produce ˙O_2_^−^ (its *E*_CB_ is more positive than −0.33 eV). The Z-scheme heterojunction of BiFeO_3_/Ag/g-C_3_N_4_ is formed to achieve the photocatalytic degradation of MB. Given that the *E*_CB_ of g-C_3_N_4_ (−1.27 eV) is more negative than that of O_2_/˙O_2_^−^ (−0.33 eV *vs.* NHE), it can capture adsorbed oxygen to form ˙O_2_^−^ radicals. The *E*_VB_ of BiFeO_3_ (−2.73 eV) is more positive than the reduction potential of H_2_O/˙OH (2.38 eV *vs.* NHE), and ˙OH radicals are created.^70^ The photogenerated electrons from the CB of BiFeO_3_ can rapidly transfer to g-C_3_N_4_ through plasmonic Ag, and combine with the holes from the VB of g-C_3_N_4_. Consequently, the electrons from the CB of g-C_3_N_4_ and the holes from the VB of BiFeO_3_ are effectively separated. The MB in solution is oxidized and degraded in the presence of ˙OH and ˙O_2_^−^ to CO_2_, H_2_O, mineral acids.^71^ The possible reactions are as follows:8
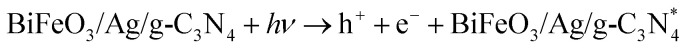

9 H_2_O + h^+^ → ˙OH + H^+^

10 O_2_ + e^−^ → ˙O_2_^−^

11 ˙OH + MB → degraded product

12 ˙O_2_^−^ + MB → degraded product

## 4. Conclusion

In summary, a Z-scheme heterojunction of 1D/0D/2D BiFeO_3_/Ag/g-C_3_N_4_ was constructed to enhance the photocatalytic activity in degradation of organic dyes under visible light irradiation. The structural parameters, optical properties, photocatalytic dye degradation, and dielectric properties of the materials were assessed by XRD, XPS, BET, SEM, EDX, UV-vis DRS, PL, electrochemical and photocatalytic degradation testing. The EPR and free radical scavenging experiments confirmed the active species (˙OH and ˙O_2_^−^) for the decomposition of MB, demonstrating the possible photocatalytic mechanism. This study provides a feasible way for improving the visible light degradation of dyes in wastewater, and has significant reference for the design and synthesis of novel Z-scheme heterojunction photocatalysts with multidimensional contact interfaces.

## Author contributions

Donghai Li: investigation, writing – original draft, data curation and validation; Yunrui Xu: visualization, investigation; Shilin Zhang: visualization, investigation; Linping Wang: writing – review & editing, writing – original draft, supervision, investigation, funding acquisition.

## Conflicts of interest

We declare that there is no conflict of interest to this work.

## Supplementary Material

RA-015-D5RA04825G-s001

## Data Availability

The data supporting this article have been included as part of the SI. No additional datasets were generated or analyzed during this study. Supplementary information: The authors declare that the data supporting the findings of this study are available within the paper and its SI. Isotherm linear plots, dark reaction kinetic curves, effects of Ag and g-C_3_N_4_ mass fractions on efficiency, LC-MS spectra, photocatalytic stability and comparison table of photocatalytic degradation. See DOI: https://doi.org/10.1039/d5ra04825g.
