# Supplementary material for: Construction of a 1D/0D/2D BiFeO3/Ag/g-C3N4 Z-scheme heterojunction for enhanced visible light photocatalysis of methylene blue
Source: RSC Adv. 2025 Sep 2;15(38):31508–21. doi: 10.1039/d5ra04825g (PMC12402981; doi:10.1039/d5ra04825g)
Supplement: RA-015-D5RA04825G-s001 [file RA-015-D5RA04825G-s001.pdf]

Supplementary Information (SI) for RSC Advances.
This journal is © The Royal Society of Chemistry 2025

Supplementary Material

Construction of a 1D/0D/2D $BiFeO_3$/Ag/g-C_3N_4 Z-scheme heterojunction for enhanced visible light photocatalysis of methylene blue

Donghai Li, Yunrui Xu, Shilin Zhang, Linping Wang*

College of Chemical Engineering, Qinghai University, Xining-810016, China

*Corresponding author E-mail: wanglp@qhu.edu.cn

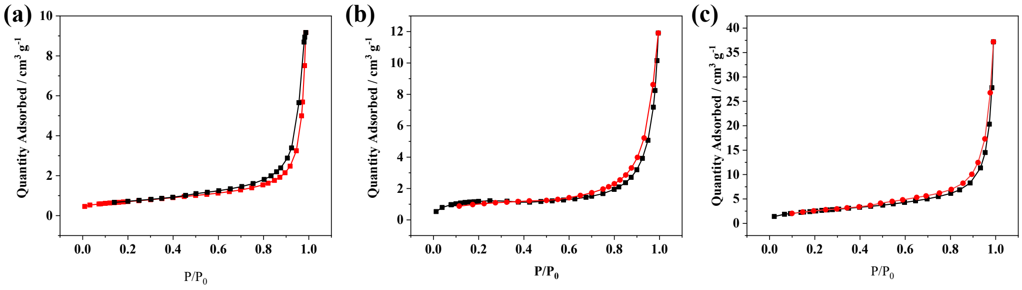

Fig. S1. Isotherm Linear plots of (a) g-C_3N_4, (b) $BiFeO_3$/$Ag_{0.05}$ and (c) ($BiFeO_3$/$Ag_{0.05}$)/(g-C_3N_4)$_{0.3}$.

Table.S1. The specific surface area of the synthesized photocatalysts

Photocatalyst	Specific Surface Area (m^2/g)
g-C_3N_4	3.8095
$BiFeO_3$/$Ag_{0.05}$	6.0673
($BiFeO_3$/$Ag_{0.05}$)/(g-C_3N_4)$_{0.3}$.	15.2139

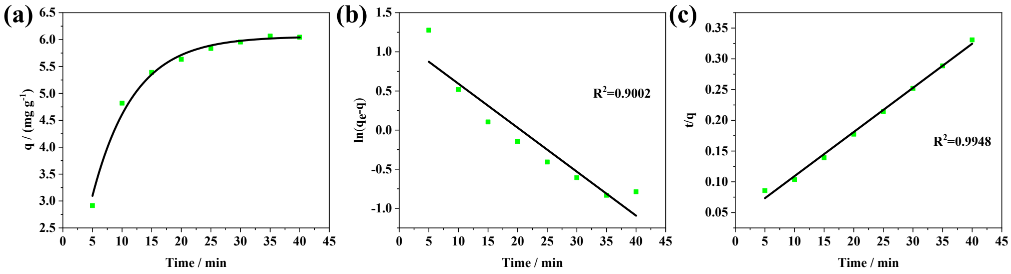

Fig. S2. (a) The dark reaction kinetics curve of MB by g-C_3N_4. The fits to pseudo-first-order (b) and pseudo-second-order (c) kinetic models.

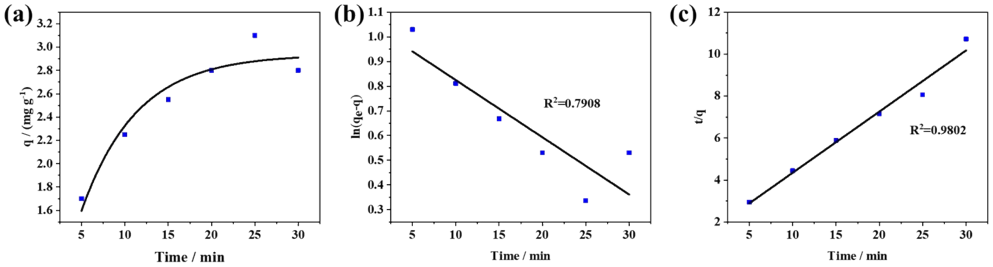

Fig. S3. (a) The dark reaction kinetics curve of MB by $(BiFeO_3/Ag_{0.05})/(g\text{-}C_3N_4)_{0.3}$. The fits to pseudo-first-order (b) and pseudo-second-order (c) kinetic models.

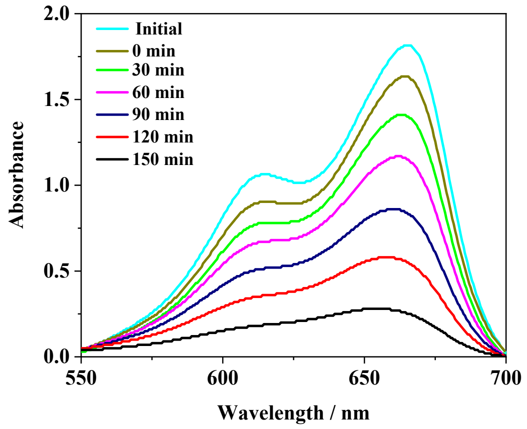

Fig. S4. Ultraviolet spectra of $(BiFeO_3/Ag_{0.05})/(g\text{-}C_3N_4)_{0.3}$ for degradation of MB.

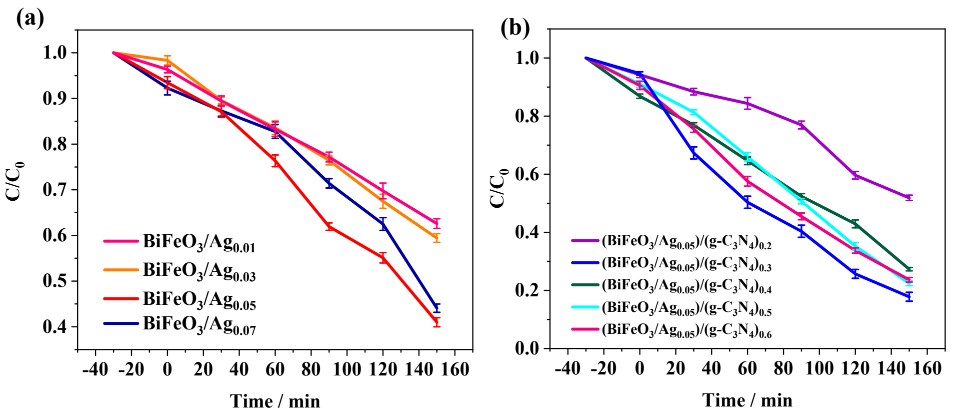

Fig. S5. Effect of catalytic efficiency on the degradation of MB: (a) different mass fractions of Ag in $BiFeO_3$/Ag, (b) mass fractions of g-C_3N_4 and $BiFeO_3/Ag_{0.05}$.

Table.S2 Comparison of photocatalytic degradation of pollutants with earlier reports.

Photocatalyst	pollutant	Catalyst dosage (mg)	Concentration of pollutant （mg / L）	Efficiency (%)	Ref.
$g\text{-}C_3N_4/Ag_2CO_3/GO$	TC	30	20	81.6	1
$g\text{-}C_3N_4$/UiO-66-NH_2/CdS	TC	50	20	83.0	2
$Ag/AgVO_3/g\text{-}C_3N_4$	CIP	100	10	82.6	3
$MoS_2/g\text{-}C_3N_4/Co_3O_4$	RhB	50	5	82.1	4
2 % GO-$g\text{-}C_3N_4$	MB	50	10	67.3	5
$Bi_2S_3/BiFeO_3$	TC	30	20	74	6
$BiFeO_3$/N-GO	CR	10	20	55	7
$(BiFeO_3/Ag_{0.05)}/(g\text{-}C_3N_4)_{0.3}$	MB	10	10	84.6	This work

GO: graphene oxide, TC: tetracycline, CIP: ciprofloxacin, RhB: rhodamine B, MB: methyl blue, CR: congo red

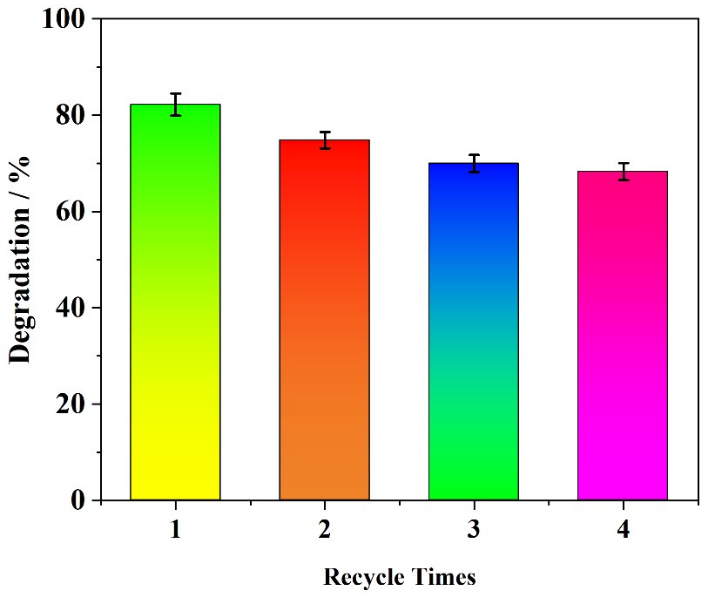

Fig. S6. Photocatalytic stability of $(BiFeO_3/Ag_{0.05})/(g\text{-}C_3N_4)_{0.3}$.

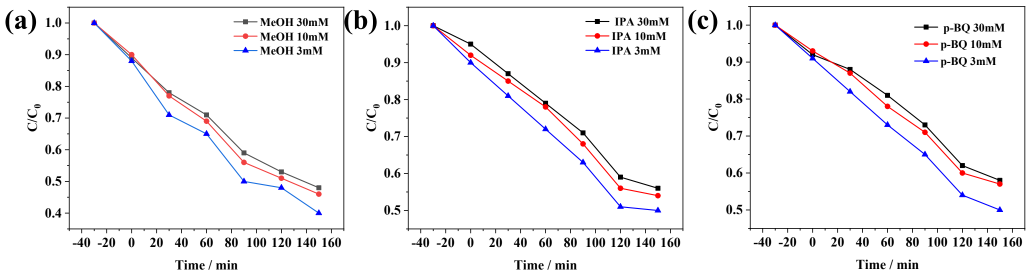

Fig. S7. The effect of different concentrations of (a) MeOH, (b) IPA, and (c) p-BQ on photocatalytic 10 mg/L MB.

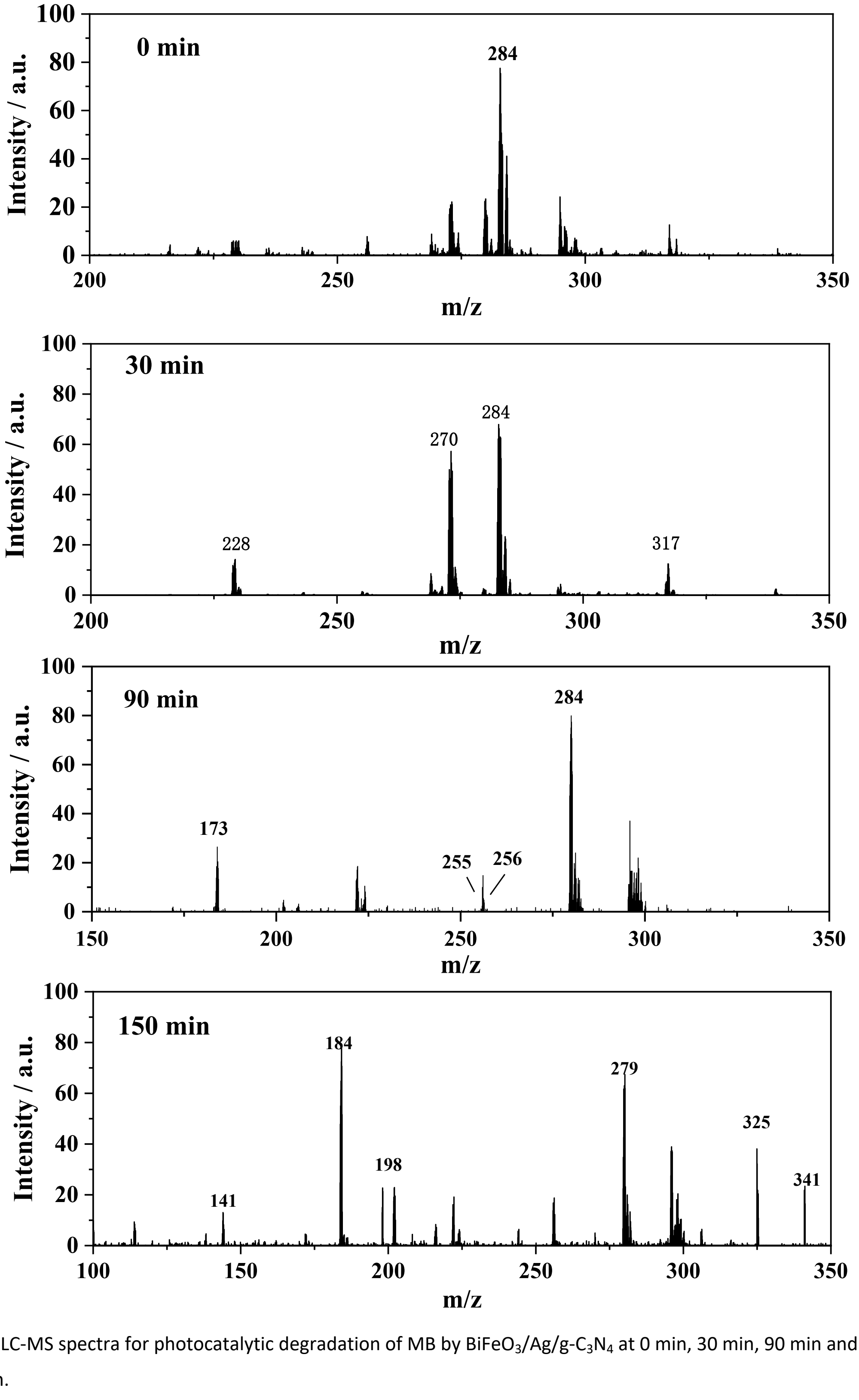

Fig. S8. LC-MS spectra for photocatalytic degradation of MB by $BiFeO_3/Ag/g\text{-}C_3N_4$ at 0 min, 30 min, 90 min and 150 min.

References

1 H. Liu, C. Liang, C. Niu, D. Huang, Y. Du, H. Guo, L. Zhang, Y. Yang and G. Zeng, Facile assembly of g-C_3N_4/Ag_2CO_3/graphene oxide with a novel dual Z-scheme system for enhanced photocatalytic pollutant degradation, *Appl Surf Sci*, 2019, **475**, 421-434.

2 H. Zhang, J. Li, X. He and B. Liu, Preparation of a g-C_3N_4/UiO-66-NH_2/CdS photocatalyst with enhanced visible light photocatalytic activity for tetracycline degradation, *Nanomaterials*, 2020, **10**, 1824-1838.

3 M. F. R. Samsudin, C. Frebillot, Y. Kaddoury, S. Sufian and W. Ong, Bifunctional Z-Scheme Ag/$AgVO_3$/g-C_3N_4 photocatalysts for expired ciprofloxacin degradation and hydrogen production from natural rainwater without using scavengers, *Journal of Environmental Management*, 2020, **270**, 110803-110812.

4 Y. Zhuang, S. Meng, X. Yang, D. Guo, D. Zhang, T. Peng, Y. Li and J. Li, Construction of amorphous-crystalline MoS_2/g-C_3N_4/Co_3O_4 ternary heterojunctions with flower-like structure for enhanced visible-light photocatalytic degradation performance, *Appl Surf Sci*, 2025, **699**, 163188-163199.

5 H. Zhang, H. Xu, R. Li, L. Quan, C. Zhan, P. Han, Y. Liu and Y. Tong, g-C_3N_4 coupled with GO accelerates carrier separation via high conductivity for photocatalytic MB degradation, *Colloids and Surfaces A: Physicochemical and Engineering Aspects*, 2024, **703**, 135311-135324.

6 Y. Ma, P. Lv, F. Duan, J. Sheng, S. Lu, H. Zhu, M. Du and M. Chen, Direct Z-scheme Bi_2S_3/$BiFeO_3$ heterojunction nanofibers with enhanced photocatalytic activity, *J Alloys Compd*, 2020, **834**, 155158-155170.

7 P. Li, Q. Chen, Y. Lin, G. Chang and Y. He, Effects of crystallite structure and interface band alignment on the photocatalytic property of bismuth ferrite/(N-doped) graphene composites, *J Alloys Compd*, 2016, **672**, 497-504.
